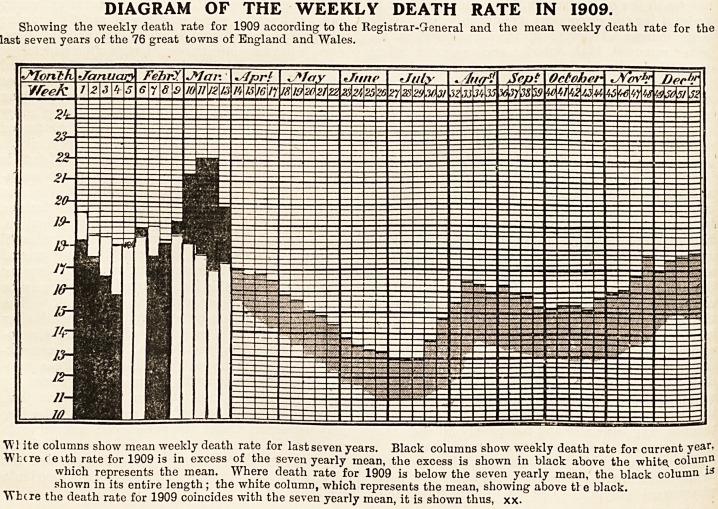# Giemsa's Method of Staining the Treponema Pallidum
*From "La Syphilis." Levaditi et Roché. Masson & Cie.


**Published:** 1909-05-01

**Authors:** 


### DERMATOLOGY.

### GIEMSA'S METHOD OF STAINING THE TREPONEMA PALLIDUM.*

*Slow Method.*—To secure good preparations it is essential that the smear be carefully made and well fixed before staining. The smear may be made either on a slide or on a cover-glass. A small drop of the clear fluid which exudes after scraping the surface of a chancre should be placed on the slide and spread out in a thin and uniform layer. If there are any solid particles, these may be crushed between two cover-glasses, after moistening with a little normal saline solution. The best method of fixation is by alcohol. After drying the smear in the air the slides or cover-glasses are plunged into a beaker containing alcohol, covered up, and left for half an hour or longer.

The stain is prepared by Grübler under the name, "Giemsa's Solution for Romanowsky Staining." Its exact composition is as follows:

| | |
|---|---|
| Azur II.—Eosin ... ... ... | 3 grammes |
| Azur II. ... ... ... ... | .8 grammes |
| Glycerine (Merck, chemically pure); and Methylic alcohol | (of each) 250 c.c. |

Just before use, 15 drops of this are mixed with 10 c.c. of water in a Petri dish. Into this all preparations are put for several hours. They are then washed in water, dried on filter paper, and mounted in balsam.

*Rapid Method.*—The smears (as thin as possible) are made on the slide, not on a cover-glass. If the preparations have been made some little time simple drying in the air is sufficient to fix them; but fresh specimens should be fixed by passing the slide three times through the flame of a spirit lamp or of a half-turned-on Bunsen burner.

Ten drops of the Giemsa stain are put into a test-tube containing 10 c.c of distilled water, taking care to avoid the slightest trace of acid in the water. To avoid the formation of precipitates the vessels used must be quite clean, and the Giemsa stain must be added to the water drop by drop, while the test-tube is gently shaken. The dilution should be made just before use. The staining powers are intensified by the addition of 5 to 10 drops of a 1-in-1,000 solution of carbonate of soda.

The slide is held in forceps, and a few drops of staining mixture are placed over the smear. It is then gently warmed over the spirit lamp flame for a few seconds, until vapour arises, but short of boiling. The solution is then thrown off; a fresh lot is put on, warmed as before, and thrown off. A third lot is dropped on, and warmed gently for one to two minutes. The slide is then washed in water, dried on filter paper, and mounted in balsam.

The treponema is coloured a reddish violet. The first method is recommended for the study of details of structure of the parasite and its relationship with the cellular elements; the latter for diagnosis.

* From "La Syphilis." Levaditi et Roché. Masson & Cie.

### DIAGRAM OF THE WEEKLY DEATH RATE IN 1909.

Showing the weekly death rate for 1909 according to the Registrar-General and the mean weekly death rate for the last seven years of the 76 great towns of England and Wales.

White columns show mean weekly death rate for last seven years. Black columns show weekly death rate for current year.
Where death rate for 1909 is in excess of the seven yearly mean, the excess is shown in black above the white column which represents the mean. Where death rate for 1909 is below the seven yearly mean, the black column is shown in its entire length; the white column, which represents the mean, showing above the black.
Where the death rate for 1909 coincides with the seven yearly mean, it is shown thus, XX.

## Figures and Tables

**Figure f1:**